# A case report of ChAdOx1 nCoV-19 vaccine–associated encephalitis

**DOI:** 10.1186/s12883-021-02517-w

**Published:** 2021-12-13

**Authors:** Junko Takata, Simon M. Durkin, Solomon Wong, Michael S. Zandi, Josephine K. Swanton, Tumena W. Corrah

**Affiliations:** 1 Department of Infectious Diseases and Tropical Medicine, Northwick Park Hospital, London North West University Healthcare NHS Trust, Watford Road, Harrow, HA1 3UJ UK; 2 Harrow Crisis Team, Central and North West London NHS Foundation Trust, Northwick Park Hospital, Harrow, London, HA1 3UJ UK; 3 National Hospital for Neurology and Neurosurgery, University College London Hospitals NHS Foundation Trust, Queen Square, London, WC1N 3BG UK; 4 Department of Neurology, Northwick Park Hospital, London North West University Healthcare NHS Trust, Watford Road, Harrow, HA1 3UJ UK

**Keywords:** COVID-19, Prevention & control (PC), Encephalitis, SARS-CoV-2, Vaccination, Adverse effects (AE)

## Abstract

**Background:**

Vaccination against COVID-19 continues apace, but side-effects, both common and severe, continue to be reported. We report here the first published case of COVID-19 vaccine-related encephalitis.

**Case presentation:**

A young woman presented with acute neuropsychiatric symptoms following recent ChAdOx1 nCoV-19 vaccination. Extensive investigation did not identify alternative causes.

**Conclusions:**

This difficult case is here described, including presentation, investigation, and management. Further study on neuropsychiatric side-effects of COVID-19 vaccination, including investigation as to whether there may be a causal link, is required.

## Key points

- Management of acute neuropsychiatric presentations in young patients is challenging.
- The temporal association, together with the clinical syndrome and lack of other causes on investigation suggests a possible COVID-19 vaccine-related encephalitis by Graus criteria.
- Further research to determine causation of neurological symptoms after COVID-19 vaccination is required; in particular, determination of those that may be coincidental, and, where causation is possible, mechanisms, biomarkers, and case management.

## Background

Vaccination against SARS-CoV-2 has proceeded apace in 2021 with several vaccine candidates. Reports of side effects, both common and rarer, are emerging. Here we present the first published case of ChAdOx1 nCoV-19 vaccine-associated encephalitis and the challenges inherent in our patient’s management.

## Case presentation

A 22-year-old woman of Middle Eastern origin, born in Iran and recently living in Western Europe, presented to a UK hospital with a three-week history of intermittent frontal headache and fatigue a few days after she received her second dose of AstraZeneca ChAdOx1 nCoV-19 vaccine. These symptoms did not respond to paracetamol and progressed on to an acute two-day history of confusion and hallucinations (visual and tactile). She reported seeing “disco balls” and experiencing someone touching her skin. She had non-syndromic retinitis pigmentosa but no other medical co-morbidities, and there was no personal or family history of psychiatric illness. She was a non-smoker and non-drinker, and enjoying undergraduate studies at university.

On admission, she had one recorded fever of 38ºC, but otherwise-normal observations (HR 91, BP 123/81, RR 18, O_2_ saturations 100% on air). She was alert but disorientated to time, person and place, and agitated with a labile affect; she complained of auditory and visual hallucinations – for example, that the room was on fire – and delusions that were often hyper-religious in nature. She was also seen to be gesturing to the air as if responding to unseen stimuli. On examination, excepting very restricted visual fields, there was no focal neurology, meningism or photophobia, and cardio-respiratory and abdominal examination was normal. Initial investigations revealed Hb 126 g/L, WCC 6.6 × 10^9^/L (neutrophils 4.6 × 10^9^/L), CRP 1.1 mg/L (reference range < 5 mg/L), normal renal and liver function, platelets 275 × 10^9^/L, and D-dimer of 1,240 ng/mL (reference range < 500 ng/mL). Chest X-ray was unremarkable, urine microscopy and culture negative, and PCR for SARS-CoV-2 and other respiratory viruses was negative (see Table 1). She was empirically commenced on intravenous ceftriaxone 2 g BD and acyclovir 800 mg TDS for possible meningo-encephalitis pending lumbar puncture (LP). She was transferred to the Infectious Diseases ward.

Initially she required rapid pharmacological tranquilisation with lorazepam and haloperidol to manage extreme agitation. CT head and CT venogram were unremarkable. Initial LP, performed 2 days after the fever, revealed an opening pressure of 30 cm H_2_O and WCC 6 cells/µL (100% lymphocytes) with negative microbiology and virology (see Table 2) on cerebrospinal fluid (CSF) analysis. She had no further recorded fevers throughout her admission and antibiotics and antivirals were stopped.

**Table 1:** Summary of investigations

Investigation	Result
**Microbiology and virology**	
SARS-CoV-2 PCR (multiple)	Negative
Extended respiratory viral PCR ^a^	Negative
Urine microscopy & culture	Negative
HIV-1/-2 serology (serum)	Negative
Hepatitis B & C serology (serum)	Negative
Syphilis serology (serum)	Negative
Measles, mumps, rubella serology (serum)	Immune
Cryptococcal antigen (serum)	Negative
Stool norovirus & enterovirus PCR	Negative
* Borrelia burgdorferi* (Lyme) serology (serum)	Negative
* Leptospira* spp. IgG & IgM (EIA) (serum)	Negative
Tick borne encephalitis virus IgG (IF) (serum)	Negative
West Nile Virus IgG & IgM (ELISA) (serum)	Negative
**Urinary tests**	
Urinary porphyrins	Negative
Urinary drugs of abuse^b^	Negative
**Serum**	
B12, folate, TSH	Normal
Vitamin D	Deficient (31 nmol/L), replaced to 68 nmol/L
ANA, ENA^c^	Negative
Rheumatoid factor, anti-CCP Ab	Negative
Anti-cardiolipin Ab, dsDNA Ab	Negative
Anti-GAD Ab	Negative
Anti-VGCC Ab	Negative
Anti-CASPR2 Ab	Negative
Anti-LG-1 Ab	Negative
DPPX immunoglobulins	Negative
Anti-neuronal Ab panel (Hu, Ri,GAD, amphiphysin, CV2, Ma1, Ma2, SOX-1, Tr-DNER)^d^	Negative
Anti-NMDA receptor Ab	Negative
Anti-GABA-A/-B Ab	Negative
Anti-AMPA Ab	Negative
Aquaporin-4 Ab	Negative
Anti-MOG Ab	Negative
Serum oligoclonal bands (paired)	Negative (CSF positive)
**CSF**	
HSV type I & II DNA	Negative
VZV DNA	Negative
Enterovirus RNA	Negative
CMV DNA	Negative
EBV DNA	Negative
HHV-6 DNA	Negative
AAFB microscopy/culture & TB PCR	Negative
Anti-NMDA receptor Ab	Negative
Anti-LG-1 Ab	Negative
Anti-CASPR2 Ab	Negative
CSF oligoclonal bands (paired)	Positive (IgG band) (serum negative)
Immunophenotyping	Not performed (normal WCC on third CSF sample)

^a^Incorporating influenza A/B, RSV, parainfluenza type 1–4, metapneumovirus, adenovirus, rhinovirus

^b^Incorporating testing for: Opiates; opioids; cannabinoids; cocaine; amphetamines; MDMA; benzodiazepines; ketamine

^c^Incorporating extractable nuclear antibodies, ribonucleoprotein antibodies, Smith antibodies, Ro antibodies, La antibodies, Jo-1 antibodies, Scl-70 antibodies, centromere antibodies

^d^Incorporating antibodies to Purkinje cells, anti-Tr antibodies, other cerebellar cells, IgG white matter (myelin), anti-Hu, anti-Yo, anti-Ri, anti-Ma-1, anti-Ma-2, anti-CV2 (CRMP-5), anti-amphiphysin, anti-Zic-4, anti-Sox 1, anti-Tr antibody DNER antigen, anti-GAD immunoblotting

**Table 2:** Summary of lumbar puncture cerebrospinal fluid (CSF) results

	**Day 2**	**Day 7**	**Day 14**
Opening pressure	30 cm H_2_O	-	15 cm H_2_O
WCC (cells/µL)	6 (100% lymph)	18 (100% lymph)	< 1
RBC (cells/µL)	< 1	< 1	22
Organisms	Nil/NO GROWTH	Nil/NO GROWTH	Nil/NO GROWTH
Virology (PCRs)	HSV/VZV/enterovirus NEG	HSV/VZV/enterovirus/CMV NEG	HSV/VZV/enterovirus/CMV/EBV/HHV-6 NEG
TB (AAFB/PCR)	-	NEG	NEG
Protein (g/L)	0.27	0.25	0.25
Glucose (mmol/L)	3.6	2.8 (plasma 4.2)	3.0
Lactate (mmol/L)	2.9	1.8	1.6
Cytology	-	Few/mature lymphocytes	Lymphocytes, no atypia

Reference ranges: WCC < 5 cells/µL; protein 0.15—0.45 g/L; glucose 1.8—4.7 mmol/L; lactate 1.0—2.9 mmol/L

Her psychotic symptoms continued in the second week, and due to dystonia, the haloperidol was switched to regular olanzapine, titrated to a maximum of 7.5 mg/day, and PRN promethazine. MRI brain on day 7 was normal, and EEG on day 8 was also normal with no evidence of seizure activity or encephalopathy. CT imaging of the abdomen was not performed due to the patient’s age. Abdominal and pelvic ultrasound was therefore conducted to exclude ovarian teratoma as a secondary cause of autoimmune encephalopathy, and this was also normal. A second LP on day 7 showed abnormal WCC of 18 cells/µL (100% lymphocytes) and her CSF was positive for IgG oligoclonal bands (with negative paired serum oligoclonal bands). Serum and CSF was sent to UK national neuro-immunology and infection reference laboratories in Oxford and London, and Porton Down, respectively, with a variety of specialist tests undertaken (Table 1) based on the clinical history. Cell surface antibodies were tested using commercial kit-based assays. Her case was discussed in the encephalitis multidisciplinary team (MDT) meeting at the National Hospital for Neurology & Neurosurgery, UK, and a possibility of autoimmune encephalitis secondary to the COVID-19 vaccine was considered given the temporal association and CSF pleocytosis (possible autoimmune encephalitis by Graus criteria [1]). However, a decision was made to avoid giving any steroids or other immunomodulatory therapy given the possibility of an infective cause pending repeat LP and given early improvements in her clinical state.

In the third week her symptoms improved with no further hallucinations and a Montreal Cognitive Assessment (MoCA) score of 22/30, and she was weaned off olanzapine. She had a third lumbar puncture on day 14, second EEG on day 22, and a second MRI on day 23, which were all normal. Other investigations were undertaken (see Table 1). She had a mild relapse in her delirium with psychotic symptoms, with simple auditory and visual hallucinations (seeing “green lights” and hearing “the sound of coins pouring into a dish”, hyper-religiosity, continuous praying and labile mood). Neurological examination remained normal, with no unresponsive episodes, witnessed involuntary movements, seizure activity, or evidence of aura. These features stabilised with resumption of olanzapine (titrated to 5 mg twice-daily), and she was successfully discharged with ongoing follow-up with Neurology and Psychiatry. The case was notified to the national COVID-19 vaccine adverse event reporting scheme.

One month after discharge she remains on low dose olanzapine and is functionally well with independent activities of daily living. However, her family report that she has not recovered back to her pre-morbid state. She is quieter and more withdrawn now, and still has residual psychotic symptoms of occasionally seeing green lights and praying more than before.

## Discussion and conclusion

This case presented substantial challenges for the treating medical team, not least due to the patient’s distress during her illness and the diagnostic conundrum presented. Neurology opinion was sought locally with input from the regional referral centre. Extensive involvement of Psychiatry colleagues was also paramount in management.

The presentation was of a limbic encephalitis. Neuroborreliosis was considered, but with absence of epidemiological risk such as travel to endemic areas within the UK or abroad, negative serum serology, normal brain imaging, and resolution of the CSF pleiocytosis, it was felt that this was unlikely; and there was an extensive workup for common known viruses. The variety of investigations outlined above aimed to identify autoimmune and paraneoplastic causes. Shin et al*.* provide further discussion of aetiology and management [2].

Isolated reports exist of anti-NMDA receptor–associated encephalitis following Japanese encephalitis, H1N1, and diphtheria, tetanus, pertussis and polio vaccinations, with plausible biochemical evidence of causality [3].

It is known that psychiatric illness, delirium, encephalopathy and encephalitis may complicate COVID-19 [4, 5], and potential biomarkers exist to support these diagnoses alongside imaging changes [6]. In this patient, however, multiple negative SARS-CoV-2 PCR tests made this diagnosis less likely, although an asymptomatic COVID-19infection around the time of vaccination cannot be excluded.

To our knowledge, there have not yet been any published case reports of encephalopathy, encephalitis, or neuropsychiatric syndromes following vaccination against COVID-19. Data from clinical trials until early in the global vaccination campaign was incomplete but overall reassuring [7]. The initial ChAdOx1 nCoV-19 Oxford vaccine trial​ was paused following a case of transverse myelitis that later was confirmed to be multiple sclerosis (MS), but there were no documented cases of encephalitis or psychosis in 12,408 participants vaccinated between April and December 2020 [8]. Yellow Card adverse incident reports to the UK Medicines & Healthcare Regulatory Agency (MHRA) from 4 January 2021 to 16 June 2021 list 32 cases of ‘encephalitis’​, 4 cases of ‘noninfective encephalitis’​, and 3 cases of ‘autoimmune encephalopathy’ temporally associated with ChAdOx1 nCoV-19 administration [9]. Various psychiatric conditions are also listed. It is not yet possible to analyse comprehensively the reports presented.

Whilst temporal relationship is not indicative of causation, the clinical syndrome, coupled with evidence of inflammatory changes in cerebrospinal fluid, and lack of evidence of an infective or immune-mediated cause, would plausibly fit with vaccine-induced encephalitis and encephalopathy. Determination of causality in this relationship is not currently possible as no specific test yet exists [10]. On this basis, a claim for litigation for a case of ‘acute encephalopathy’ reported in a ChAdOx1 nCoV-19 trial participant in India was dismissed as no evidence could be found of a causal link [11].

There is an association of psychosis and neurological complications after COVID-19, including high rates of stroke (such as in the study by Taquet et al*.* [12]), but overwhelming evidence suggests that vaccination is safe and effective, and that such risks are minimal compared to COVID-19 infection in the unvaccinated.

In summary, we believe that the patient presented in this case may have experienced a rare side-effect of COVID-19 vaccination, in the absence of another identifiable cause. Uncertainty remains, and there is no current definitive proof of causality for the patient’s symptoms and signs. Further work is urgently required to clarify this association and compare data with pre-pandemic disease incidence, to determine the mechanism involved, and to formulate appropriate treatment strategies. Encephalitis is a recognised complication of COVID-19 and at far greater rates than our sole case of uncertain causation with vaccination.

### Patient perspective

My illness started as a headache, slowly becoming throbbing and waking me at night. Nineteen days after receiving my second vaccine dose, I started to hallucinate right after feeling euphoric. I then hallucinated silhouettes running past me in slow motion. We initially thought it could be cerebral venous thrombosis. The CT scan turned out to be normal. The first two weeks I spent at the hospital were very difficult: I only remember my hallucinations and don’t recall anything else (including the staff). I also remember being convinced that I had schizophrenia and was very distraught.

However, I was beginning to recover towards the third week of my stay and was no longer hallucinating frightening things. The last two weeks I spent at the hospital were wonderful; I remember the staff and they took excellent care of me. My recovery would’ve taken longer if it wasn’t for the staff and their caring and considerate attitude. The time I spent with the nurses allowed me to gain an insight into what it takes to be a nurse, and I hope to apply for further study next year. Overall, my experience at the hospital was very positive and that was made possible by the staff.

## Data Availability

All data generated or analysed during this study are included in this published article.

## Abbreviations

BP: Blood pressure
CSF: Cerebrospinal fluid
CT: Computed tomography
EEG: Electroencephalogram
HR: Heart rate
LP: Lumbar puncture
MoCA: Montreal Cognitive Assessment
MRI: Magnetic resonance imaging
MS: Multiple sclerosis
PCR: Polymerase chain reaction
RR: Respiratory rate
SARS-CoV-2: Severe Acute Respiratory Syndrome Coronavirus 2
